# Prevalence and molecular characterization of *Wolbachia* in field-collected *Aedes albopictus*, *Anopheles sinensis*, *Armigeres subalbatus*, *Culex pipiens* and *Cx*. *tritaeniorhynchus* in China

**DOI:** 10.1371/journal.pntd.0009911

**Published:** 2021-10-28

**Authors:** Yi Yang, Yifan He, Guoding Zhu, Jilei Zhang, Zaicheng Gong, Siyang Huang, Guangwu Lu, Yalan Peng, Yining Meng, Xiaoli Hao, Chengming Wang, Jie Sun, Shaobin Shang

**Affiliations:** 1 Jiangsu Co-innovation Center for Prevention and Control of Important Animal Infectious Diseases and Zoonoses; College of Veterinary Medicine, Yangzhou University, Yangzhou, China; 2 International Corporation Laboratory of Agriculture and Agricultural Products Safety, Yangzhou University, Yangzhou, China; 3 National Health Commission Key Laboratory of Parasitic Disease Control and Prevention, Jiangsu Provincial Key Laboratory on Parasite and Vector Control Technology, Jiangsu Institute of Parasitic Diseases, Wuxi, China; 4 Division of Gastroenterology and Hepatology, Department of Medicine, University of Illinois at Chicago, Chicago, Illinois, United States of America; 5 Department of Pathobiology, College of Veterinary Medicine, Auburn University, Auburn, Alabama, United States of America; 6 Shenzhen Academy of Inspection and Quarantine Sciences, Shenzhen, China; Texas Biomedical Research institute, UNITED STATES

## Abstract

*Wolbachia* are maternally transmitted intracellular bacteria that can naturally and artificially infect arthropods and nematodes. Recently, they were applied to control the spread of mosquito-borne pathogens by causing cytoplasmic incompatibility (CI) between germ cells of females and males. The ability of *Wolbachia* to induce CI is based on the prevalence and polymorphism of *Wolbachia* in natural populations of mosquitoes. In this study, we screened the natural infection level and diversity of *Wolbachia* in field-collected mosquitoes from 25 provinces of China based on partial sequence of *Wolbachia surface protein* (*wsp*) gene and multilocus sequence typing (MLST). Among the samples, 2489 mosquitoes were captured from 24 provinces between July and September, 2014 and the remaining 1025 mosquitoes were collected month-by-month in Yangzhou, Jiangsu province between September 2013 and August 2014. Our results showed that the presence of *Wolbachia* was observed in mosquitoes of *Aedes albopictus* (97.1%, 331/341), *Armigeres subalbatus* (95.8%, 481/502), *Culex pipiens* (87.0%, 1525/1752), *Cx*. *tritaeniorhynchus* (17.1%, 14/82), but not *Anopheles sinensis* (n = 88). Phylogenetic analysis indicated that high polymorphism of *wsp* and MLST loci was observed in *Ae*. *albopictus* mosquitoes, while no or low polymorphisms were in *Ar*. *subalbatus* and *Cx*. *pipiens* mosquitoes. A total of 12 unique mutations of deduced amino acid were identified in the *wsp* sequences obtained in this study, including four mutations in *Wolbachia* supergroup A and eight mutations in supergroup B. This study revealed the prevalence and polymorphism of *Wolbachia* in mosquitoes in large-scale regions of China and will provide some useful information when performing *Wolbachia*-based mosquito biocontrol strategies in China.

## Author summary

The mosquitoes *Aedes albopictus*, *Anopheles sinensis*, *Armigeres subalbatus*, *Culex pipiens* and *Cx*. *tritaeniorhynchus* are native to China and the major vectors in the transmission of arboviruses, protozoans and nematodes. Recently, an innovative biocontrol strategy has been developed and evaluated based on the ability of *Wolbachia* to induce cytoplasmic incompatibility (CI), as well as interfere with the infection and replication of pathogens. Since the ability to induce CI largely depends on the density and diversity of *Wolbachia*, we investigated and characterized the natural infection of *Wolbachia* in above-mentioned five species of field-collected mosquitoes in 25 provinces of China. The results showed that the positive rates of *Wolbachia* infection were high in mosquitoes of *Ae*. *albopictus*, *Ar*. *subalbatus* and *Cx*. *pipiens* in large-scale regions of China and low in *Cx*. *tritaeniorhynchus* in Guizhou province. Phylogenetic analysis based on *Wolbachia surface protein* (*wsp*) gene and five multilocus sequence typing (MLST) loci indicated the high polymorphism of *Wolbachia* in *Ae*. *albopictus*, and low polymorphisms in *Ar*. *subalbatus* and *Cx*. *pipiens*. This finding contributes to the understanding of the nationwide distribution of *Wolbachia* and the potential application of this biocontrol strategy in China.

## Introduction

*Wolbachia* is a genus of Gram-negative intracellular bacteria, belonging to the *α-subphylum Proteobacteria*, which is the most widely distributed symbiotic bacteria in the world and commonly found in arthropods and some nematodes. It was first reported by Hertig and Wolbach in reproductive tissues of *Culex pipiens* in 1924 [1]. In mosquitoes and many other arthropods, *Wolbachia* infection causes sperm-egg incompatibility called cytoplasmic incompatibility (CI) that leads to a reduction in egg-hatch frequencies when a *Wolbachia*-infected male mates with uninfected females [2]. The consequence of CI provides a reproductive advantage to infected females and contributes to the rapid spread of *Wolbachia* among the host population [3].

Mosquitoes are the most important vector of numerous arthropod-borne viruses (arboviruses), for example, Zika virus, dengue fever virus, West Nile virus and Japanese encephalitis virus. In order to slow the spread of mosquito-borne diseases, insecticides have been used extensively, but with limited success. At the same time, with the abuse of insecticides, mosquitoes are becoming more and more resistant. In the recent two decades, *Wolbachia* infection was demonstrated to reduce the potential transmission of mosquito-borne diseases by shortening adult lifespan, affecting mosquito reproduction and interfering with pathogen replication [4–6]. In 2013, Bian et al. established a stable infection of *Wolbachia* strain *w*AlbB in *Anopheles stephensi*, which is one of the most important vectors of malaria. The infection of *w*AlbB conferred resistance in the mosquitoes to the human malaria parasite *Plasmodium falciparum* [7].

The major lineages of *Wolbachia* have different host specificity and type of symbiosis, and are currently clustered into 20 supergroups (A–T) according to the order of their description [8,9], while only supergroups A and B have been found in mosquitoes. Initially, *16S rRNA*-based PCR and sequencing were used for the detection and molecular characterization of *Wolbachia* [10], followed by the employment of the more variable *Wolbachia surface protein* (*wsp*) gene [11]. Subsequently, a reproducible and portable method, multilocus sequence typing (MLST), was established targeting five alleles (*gatB*, *coxA*, *hcpA*, *ftsZ* and *fbpA*) [12]. Although with limitations, it was gradually used for strain differentiation in the community of *Wolbachia* researchers. Bleidorn et al. recommended that whole-genome typing methods should be applied to the characterization of *Wolbachia* [8]. Several studies have instigated the prevalence and/or molecular characterization of *Wolbachia* in certain species of mosquitoes in China, particularly *Aedes albopictus* [13,14]. However, up to date, more than 45 species of mosquitoes have been identified in 31 provinces, distributed in the east, south, north, central, northeast, southwest and northwest regions of China. Among them, *Ae*. *albopictus*, *Armigeres subalbatus*, *Cx*. *pipiens*, *Cx*. *quinquefasciatus* and *Cx*. *tritaeniorhynchus* were most widely distributed [15]. Our knowledge of the prevalence and diversity of *Wolbachia* in mosquitoes of different species and geographic regions remains limited, especially in China. Therefore, in this study, we screened the prevalence and diversity of *Wolbachia* in approximately 3500 field-collected mosquitoes of *Ae*. *albopictus*, *An*. *sinensis*, *Ar*. *subalbatus*, *Cx*. *pipiens* and *Cx*. *tritaeniorhynchus* from 25 provinces of China with *wsp*-based real-time PCR and MLST analyses.

## Materials and methods

### Ethics statement

All experimental protocols in this study were reviewed and approved by the Institutional Animal Care and Use Committee of Yangzhou University.

### Mosquito collection

**Fig 1:**
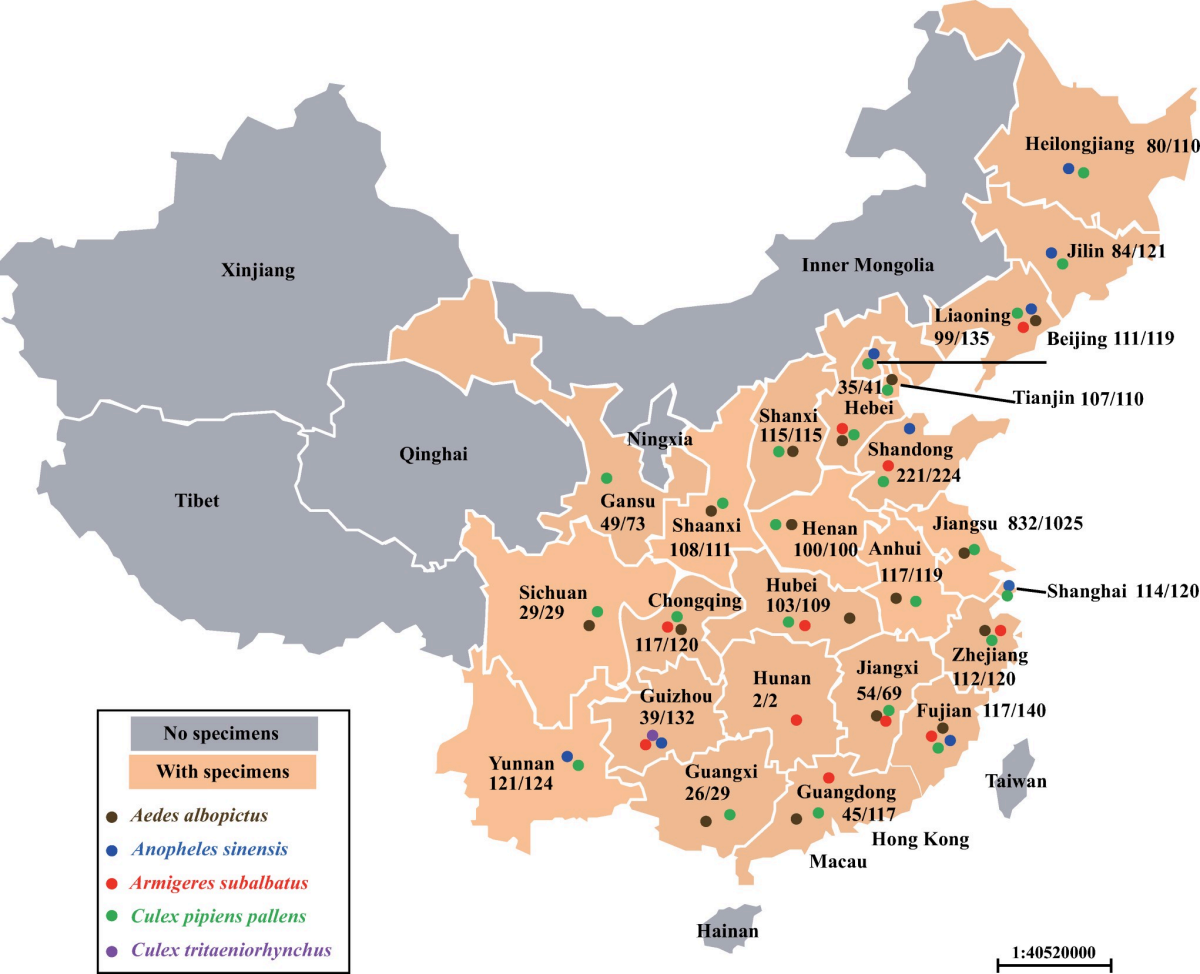
The distributions of field-collected mosquitoes in this study. A total of 3514 samples of mosquitoes’ whole bodies (convenience samples) were collected in 25 provinces of China, including *Aedes albopictus* (n = 349), *Anopheles sinensis* (n = 88), *Armigeres subalbatus* (n = 502), *Culex pipiens* (n = 2495) and *Cx*. *tritaeniorhynchus* (n = 80). Different colored dots represented different species of mosquitoes, including *Ae*. *albopictus* (brown), *An*. *sinensis* (blue), *Ar*. *subalbatus* (red), *Cx*. *pipiens* (green) and *Cx*. *tritaeniorhynchus* (purple). The numbers in the region of each province indicated the positive/ total numbers of mosquitoes.

Convenience whole-body mosquito samples (n = 3514) were trapped in summer (July and September, 2014) in 24 provinces of China, and in the whole year (September 2013 to August 2014) in Yangzhou, Jiangsu province with hand nets, for an epidemiological survey of *Rickettsia* (Fig 1) [16,17]. Once captured, the mosquitoes were immediately protected separately in sterile tubes containing 400 μL of DNA/RNA stabilization reagent (Roche, Basel, Switzerland). To prevent cross contamination, disposable gloves were changed before the collection of each mosquito. Then the samples were transported at room temperature to the laboratory for species and gender identification and DNA extraction.

### Mosquito species and gender identification

The species and genders of most mosquitoes employed in this study have been identified in our previous study [16]. The remaining samples were identified with standard morphological criteria (https://www.wrbu.si.edu/) with an ordinary microscope (Olympus, Tokyo, Japan). Each mosquito was removed from DNA/RNA stabilization reagent and placed on to a glass slide for morphological identification. In addition, the mosquitoes of each species from each province were subjected to further identification using a genomic approach targeting *mitochondrial cytochrome c oxidase subunit I* (*COI*) gene [18] (Table 1). After species and gender identification, the mosquitoes were stored at -80°C until genomic DNA extraction.

**Table 1:** Primer pairs used in this study.

Gene	Primer Sequence (5’- 3’)	Annealing Temperature	Amplicon Length (bp)	Reference
*COI*	GGTCAACAAATCATAAAGATATTGG	51°C	~650	[16]
	TAAACTTCAGGGTGACCAAAAAATCA			
*wsp*	TGGTCCAATAAGTGATGAAGAAAC	53°C	~610	[11]
	AAAAATTAAACGCTACTCCA			
*16S rRNA*	CATACCTATTCGAAGGGATAG	60°C	~438	[19]
	AGCTTCGAGTGAAACCAATTC			
*gatB*	GAKTTAAAYCGYGCAGGBGTT	54°C	~471	[12]
	TGGYAAYTCRGGYAAAGATGA			
*coxA*	TTGGRGCRATYAACTTTATAG	54°C	~487	[12]
	CTAAAGACTTTKACRCCAGT			
*hcpA*	GAAATARCAGTTGCTGCAAA	54°C	~515	[12]
	GAAAGTYRAGCAAGYTCTG			
*ftsZ*	ATYATGGARCATATAAARGATAG	54°C	~524	[12]
	TCRAGYAATGGATTRGATAT			
*fbpA*	GCTGCTCCRCTTGGYWTGAT	59°C	~509	[12]
	CCRCCAGARAAAAYYACTATTC			

### Genomic DNA extraction and molecular detection of *Wolbachia*

After thawing at room temperature for approximate 15 min, the samples were homogenized individually with Precellys 24 homogenizer (Bertin Technologies, Montigny-le-Bretonneux, France) at 5800 x rpm (two short durations of 15 s with a 15-s break in between) and subjected to DNA extraction with High-Pure PCR Template Preparation Kit (Roche, Basel, Switzerland) following the manufacturer’s instructions.

The presence of *Wolbachia* was screened using SYBR Green real-time PCR targeting a unique and highly conserved fragment of *outer surface protein* (*wsp*) gene [11], and the amplicon contained all the four hypervariable regions (HVR1-4) of *wsp*. In addition, the negative samples for *wsp* gene were further confirmed with the amplification of *16S rRNA* gene [19] (Table 1). The real-time PCR were performed in a Roche LightCycler 480 II real-time PCR platform (Roche, Basel, Switzerland) with 20-μL volumes composed of 10 μL of 2 x ChamQ Universal SYBR qPCR Master (Vazyme, Nanjing, China), 0.4 μL of forward primer, 0.4 μL of reverse primer, 2 μL of DNA template and 7.2 μL of double-distilled water (ddH_2_O) [20]. Thermal cycling consisted of a 2-min denaturation step at 94°C followed by 37 cycles of 94°C for 30 s, 53°C (*wsp*) or 60°C (*16S rRNA*) for 30 s and 72°C for 1 min, final elongation at 72°C for 10 min and final holding at 37°C. The PCR products of *wsp* (~610 bp) with different melting temperatures were gel purified using a QIAquick Gel Extraction Kit (Qiagen, Valencia, USA) and sequenced by a commercial sequencing laboratory (Tsingke Biotechnology, Tianjin, China) with sanger dideoxy sequencing. Using the gel-purified PCR products as quantitative standards, the concentrations of DNAs were determined with the Quanti-iT PicoGreen dsDNA Assay Kit (Invitrogen Corporation, Carlsbad, USA). The 10-fold dilutions were performed to give the reaction system containing 100,000, 10,000, 1,000, 100 and 10 copies of the *wsp* gene of *Wolbachia* supergroup A or B, respectively. The DNA samples of *Rickettsia bellii* and *R*. *felis* were stored in our laboratory and served as negative controls.

### Phylogenetic analysis with *wsp* and MLST

In order to investigate the molecular characterization of the mosquitoes captured from 25 provinces, one to four positive samples of each mosquito species in each province were selected to *Wolbachia* polymorphism identification with *wsp* gene sequencing and MLST targeting five alleles (*gatB*, *coxA*, *hcpA*, *ftsZ* and *fbpA*). A total of 109 *Wolbachia*-positive PCR products amplified from female or male mosquitoes of different species and different geographic regions were gel purified with the QIAquick Gel Extraction Kit (QIAGEN, Dusseldorf, Germany) and sequenced and verified by Sanger dideoxy sequencing (Sangon Biotech, Shanghai, China).

The five MLST loci were amplified with specific primers published in a previous study [12] (Table 1). PCR reaction were performed in a final volume of 25 μL containing 1 μL of DNA, 12.5 μL of 2 x Phanta Max Master Mix (Vazyme, Nanjing, China), 1 μL of forward primer, 1 μL of reverse primer and 9.5 μL of ddH_2_O. Thermal cycling consisted of a 2-min denaturation step at 94°C, followed by 37 cycles of 94°C for 30 s, optimal annealing temperature for 45 s (54°C for *gatB*, *coxA*, *hcpA*, *ftsZ* and 59°C for *fbpA*) and 72°C for 1.5 min, final elongation at 72°C for 10 min and final holding at 4°C. The PCR products (*gatB*: ~471 bp, *coxA*: ~487 bp, *hcpA*: ~515 bp, *ftsZ*: ~524 bp and *fbpA*: ~509 bp) were bi-directional sequenced by a commercial sequencing laboratory (Sangon Biotech, Shanghai, China) with sanger dideoxy method.

The sequences of partial *wsp* gene and MLST loci of *Wolbachia* obtained in this study were assembled with DNASTAR Lasergen 15.2 (DNASTAR Inc., Madison, WI) and aligned using CLUSTAL W in MEGA 7.0 (MEGA, Pennsylvania State University, University Park) along with those of *Wolbachia* strains represented different supergroups obtained from previous studies or GenBank [11,13]. A neighbor-joining phylogenetic tree was constructed using the Tamura-Nei model and the robustness of clusters was assessed by bootstrapping 1000 replicates. Maximum-likelihood phylogenetic analysis was performed to confirm the results [21]. The sequences of five MLST loci of each strain were submitted to a public database (PubMLST) to obtain allelic profiles and sequence type (ST).

### Statistical analysis

Statistical analyses were performed with the Statistica 7.0 software package (StatSoft Inc., USA). Positive rates of *Wolbachia* in different sampling time or locations were compared with the Chi-squared or Fisher’s exact test. *P*-value<0.05 indicated a significant difference.

## Results

### Mosquito species and gender identified

The identification of species and genders were performed on most mosquitoes (77.0%, 2706/3514) employed in this study, revealing the presence of five species of mosquitoes, including *Ae*. *albopictus* (n = 341, female = 258, male = 83), *An*. *sinensis* (n = 88, female = 62, male = 26), *Ar*. *subalbatus* (n = 502, female = 236, male = 266), *Cx*. *pipiens* (n = 1752, female = 992, male = 760) and *Cx*. *tritaeniorhynchus* (n = 82, female = 72, male = 10) (Table 2) with the accession numbers of OK465203~OK465358. Species identification was not performed on the samples collected in Jiangsu province from September 2013 to March 2014, because the limited number of researchers at that time.

**Table 2:** The presence and positive rates of *Wolbachia* in mosquitoes collected from 24 provinces of China.

Mosquito species	Province	Coordinate	Positive of *Wolbachia*		
			Female	Male	Total
***Ae*. *albopictus***	Anhui	31.5°N, 117.2°E	100% (2/2)	NA^a^	100% (2/2)
	Chongqing	29.3°N, 106.3°E	100% (10/10)	NA	100% (10/10)
	Fujian	26.1°N, 119.2°E	100% (1/1)	NA	100% (1/1)
	Guangdong	23.1°N, 113.1°E	80.0% (8/10)	100% (3/3)	84.6% (11/13)
	Guangxi	22.5°N, 108.2°E	100% (17/17)	NA	100% (17/17)
	Hebei	38.0°N, 114.3°E	100% (30/30)	100% (1/1)	100% (31/31)
	Henan	34.5°N, 113.4°E	100% (15/15)	NA	100% (15/15)
	Hubei	30.4°N, 114.2°E	100% (10/10)	100% (3/3)	100% (13/13)
	Jiangsu	32.0°N, 118.5°E	92.6% (25/27)	95.0% (19/20)	93.6% (44/47)
	Jiangxi	28.4°N, 115.6°E	94.7% (18/19)	NA	94.7% (18/19)
	Liaoning	41.5°N, 123.3°E	40.0% (2/5)	100% (3/3)	62.5% (5/8)
	Shaanxi	34.2°N, 108.6°E	100% (3/3)	NA	100% (3/3)
	Shandong	36.4°N, 117.0°E	100% (55/55)	100% (42/42)	100% (97/97)
	Shanxi	37.5°N, 112.3°E	100% (1/1)	NA	100% (1/1)
	Sichuan	30.4°N, 104.0°E	100% (25/25)	NA	100% (25/25)
	Tianjing	39.0°N, 117.1°E	95.0% (19/20)	100% (7/7)	96.3% (26/27)
	Zhejiang	30.2°N, 120.1°E	100% (8/8)	100% (4/4)	100% (12/12)
	Total		96.5% (249/258)	98.8% (82/83)	97.1% (331/341)
***An*. *sinensis***	Beijing	39.6°N, 116.2°E	0% (0/2)	NA	0% (0/2)
	Fujian	26.1°N, 119.2°E	0% (0/14)	0% (0/8)	0% (0/22)
	Guizhou	26.4°N, 106.4°E	0% (0/15)	0% (0/10)	0% (0/25)
	Heilongjiang	45.4°N, 126.4°E	0% (0/3)	NA	0% (0/3)
	Jilin	43.5°N, 125.2°E	0% (0/5)	NA	0% (0/5)
	Liaoning	41.5°N, 123.3°E	0% (0/15)	0% (0/6)	0% (0/21)
	Shandong	36.4°N, 117.0°E	0% (0/2)	NA	0% (0/2)
	Shanghai	31.1°N, 121.3°E	0% (0/3)	NA	0% (0/3)
	Yunnan	25.0°N, 102.4°E	0% (0/3)	NA	0% (0/3)
	Zhejiang	30.2°N, 120.1°E	NA	0% (0/2)	0% (0/2)
	Total		0% (0/62)	0% (0/26)	0% (0/88)
***Ar*. *subalbatus***	Chongqing	29.4°N, 106.3°E	97.6% (40/41)	79.0% (64/66)	97.2% (104/107)
	Fujian	26.1°N, 119.2°E	100% (77/77)	100% (38/38)	100% (115/115)
	Guangdong	23.1°N, 113.1°E	100% (5/5)	100% (28/28)	100% (33/33)
	Guizhou	26.4°N, 106.4°E	100% (16/16)	100% (9/9)	100% (25/25)
	Hebei	38.0°N, 114.3°E	100% (2/2)	NA	100% (2/2)
	Hubei	30.4°N, 114.2°E	97.1% (33/34)	95.0% (19/20)	96.3% (52/54)
	Hunan	28.1°N, 112.6°E	100% (2/2)	NA	100% (2/2)
	Jiangxi	28.4°N, 115.6°E	NA	100% (1/1)	100% (1/1)
	Liaoning	41.5°N, 123.3°E	63.6% (21/33)	100% (35/35)	82.4% (56/68)
	Shandong	36.4°N, 117.0°E	100% (4/4)	100% (1/1)	100% (5/5)
	Zhejiang	30.2°N, 120.1°E	90.9% (20/22)	97.1% (66/68)	95.6% (86/90)
	Total		93.2% (220/236)	98.1% (261/266)	95.8% (481/502)
***Cx*. *pipiens***	Anhui	31.5°N, 117.2°E	98.1% (53/54)	98.4% (62/63)	98.3% (115/117)
	Beijing	39.6°N, 116.2°E	95.2% (60/63)	94.4% (51/54)	94.9% (111/117)
	Chongqing	29.4°N, 106.3°E	NA	100% (3/3)	100% (3/3)
	Fujian	26.1°N, 119.2°E	0% (0/1)	100% (1/1)	50.0% (1/2)
	Gansu	36.0°N, 103.5°E	59.3% (35/59)	100% (14/14)	67.1% (49/73)
	Guangdong	23.1°N, 113.1°E	0% (0/68)	33.3% (1/3)	1.4% (1/71)
	Guangxi	22.5°N, 108.2°E	70.0% (7/10)	100% (2/2)	75.0% (9/12)
	Hebei	38.0°N, 114.3°E	16.7% (1/6)	50.0% (1/2)	25.0% (2/8)
	Heilongjiang	45.4°N, 126.4°E	42.2% (19/45)	98.4% (61/62)	74.8% (80/107)
	Henan	34.5°N, 113.4°E	100% (35/35)	100% (50/50)	100% (85/85)
	Hubei	30.4°N, 114.2°E	81.0% (17/21)	100% (21/21)	90.5% (38/42)
	Jiangsu	32.0°N, 118.5°E	92.6% (113/122)	82.4 (89/107)	88.2% (202/229)
	Jiangxi	28.4°N, 115.6°E	50.0% (10/20)	86.2% (25/29)	71.4% (35/49)
	Jilin	43.5°N, 125.2°E	72.2% (83/115)	100% (1/1)	72.4% (84/116)
	Liaoning	41.5°N, 123.3°E	100% (7/7)	100% (31/31)	100% (38/38)
	Shaanxi	34.2°N, 108.6°E	98.0% (97/99)	88.9% (8/9)	97.2% (105/108)
	Shandong	36.4°N, 117.0°E	98.2% (54/55)	100% (65/65)	99.2% (119/120)
	Shanghai	31.1°N, 121.3°E	96.3% (77/80)	100% (37/37)	97.4% (114/117)
	Shanxi	37.5°N, 112.3°E	100% (17/17)	100% (97/97)	100% (114/114)
	Sichuan	30.4°N, 104.0°E	100% (4/4)	NA	100% (4/4)
	Tianjing	39.0°N, 117.1°E	98.7% (76/77)	83.3% (5/6)	97.6% (81/83)
	Yunnan	25.0°N, 102.4°E	100% (27/27)	100% (94/94)	100% (121/121)
	Zhejiang	30.2°N, 120.1°E	85.7% (6/7)	88.9% (8/9)	87.5% (14/16)
	Total		80.4% (798/992)	95.7% (727/760)	87.0% (1525/1752)
***Cx*. *tritaeniorhynchus***	Guizhou	26.4°N, 106.4°E	5.6% (4/72)	100% (10/10)	17.1% (14/82)
**Unidentified**	Jiangsu	32.0°N, 118.5°E			78.2% (586/749)
**Total**					83.6% (2937/3514)

^a^ NA: not available

### Presence of *Wolbachia* in mosquitoes from 25 provinces of China

All the mosquitoes captured in 25 provinces were screened for the presence of *Wolbachia* with *wsp*-based PCR. The detection limit of this PCR was 10 copies of the *wsp* gene per reaction for both *Wolbachia* supergroups A and B, and nonspecific amplifications were not observed (S1 Fig). Overall, 83.6% of the mosquitoes (2937/3514) were positive for *Wolbachia*, including 97.1% (331/341) of *Ae*. *albopictus*, 95.8% (481/502) of *Ar*. *subalbatus*, 87.0% (1525/1752) of *Cx*. *pipiens*, 17.1% (14/82) of *Cx*. *tritaeniorhynchus* and 78.2% (586/749) of unidentified mosquitoes, while both *wsp* and *16S rRNA* genes of *Wolbachia* was unavailable to be detected in *An*. *sinensis* (n = 88). In detail, the positive rates of *Wolbachia* were 62.5~100% in *Ae*. *albopictus*, 82.4~100% in *Ar*. *subalbatus*, 1.4~100% in *Cx*. *pipiens* and 17.1% in *Cx*. *tritaeniorhynchus* sampled in different provinces of China (Table 2). The positive rate of *Wolbachia* in *Cx*. *pipiens* was significantly lower than that in *Ae*. *albopictus* and *Ar*. *subalbatus* (*P*<0.01), and significantly higher than that in *Cx*. *tritaeniorhynchus* (*P*<0.01).

### Presence of *Wolbachia* in mosquitoes in Yangzhou, Jiangsu province

From September 2013 to August 2014, a total of 1025 mosquito samples were collected in a campus in Yangzhou, Jiangsu province. The monthly positive rates of *Wolbachia* ranged from 50.8% (66/130) to 98.4% (182/185). Compared with March 2014, the mosquito samples collected from September to December (2013), April (2014) and July (2014) harbored significant lower positive rates (*P*<0.01) of *Wolbachia*. Among the samples collected from April to August (2014), of which the species and gender information is available, significant higher infection rates were found with female samples of *Cx*. *pipiens* in April (*P*<0.05) (Table 3).

**Table 3:** The presence and positive rates of *Wolbachia* in mosquitoes collected in Yangzhou, Jiangsu province between September 2013 and August 2014.

Sampling time	Daily mean Temperature (°C)	Species	Positive rate		
			Female	Male	Total
Sep 2013	20~27	NA	NA	NA	50.8% (66/130)
Oct 2013	13~23	NA	NA	NA	76.0% (225/296)
Nov 2013	7~16	NA	NA	NA	82.1% (78/95)
Dec 2013	0~9	NA	NA	NA	81.4% (35/43)
Mar 2014	6~15	NA	NA	NA	98.4% (182/185)
Apr 2014	11~20	*Cx*. *pipiens*	97.9% (47/48)	79.5% (35/44)	89.1% (82/92)
May 2014	17~27	*Cx*. *pipiens*	95.7% (22/23)	79.5% (23/23)	97.8% (45/46)
Jun 2014	20~28	*Cx*. *pipiens*	96.2% (25/26)	95.0% (19/20)	95.7% (44/46)
Jul 2014	23~30	*Ae*. *albopictus*	0% (0/1)	NA	0% (0/1)
		*Cx*. *pipiens*	76.0% (19/25)	60.0% (12/20)	68.9% (31/45)
		Total	73.1% (19/26)	60.0% (12/20)	67.4% (31/46)
Aug 2014	22~28	*Ae*. *albopictus*	96.2% (25/26)	95.0% (19/20)	95.7% (44/46)
Total	0~30				81.2% (832/1025)

^a^ NA: not available

### Molecular characterization of *Wolbachia* with *wsp* and MLST

**Fig 2:**
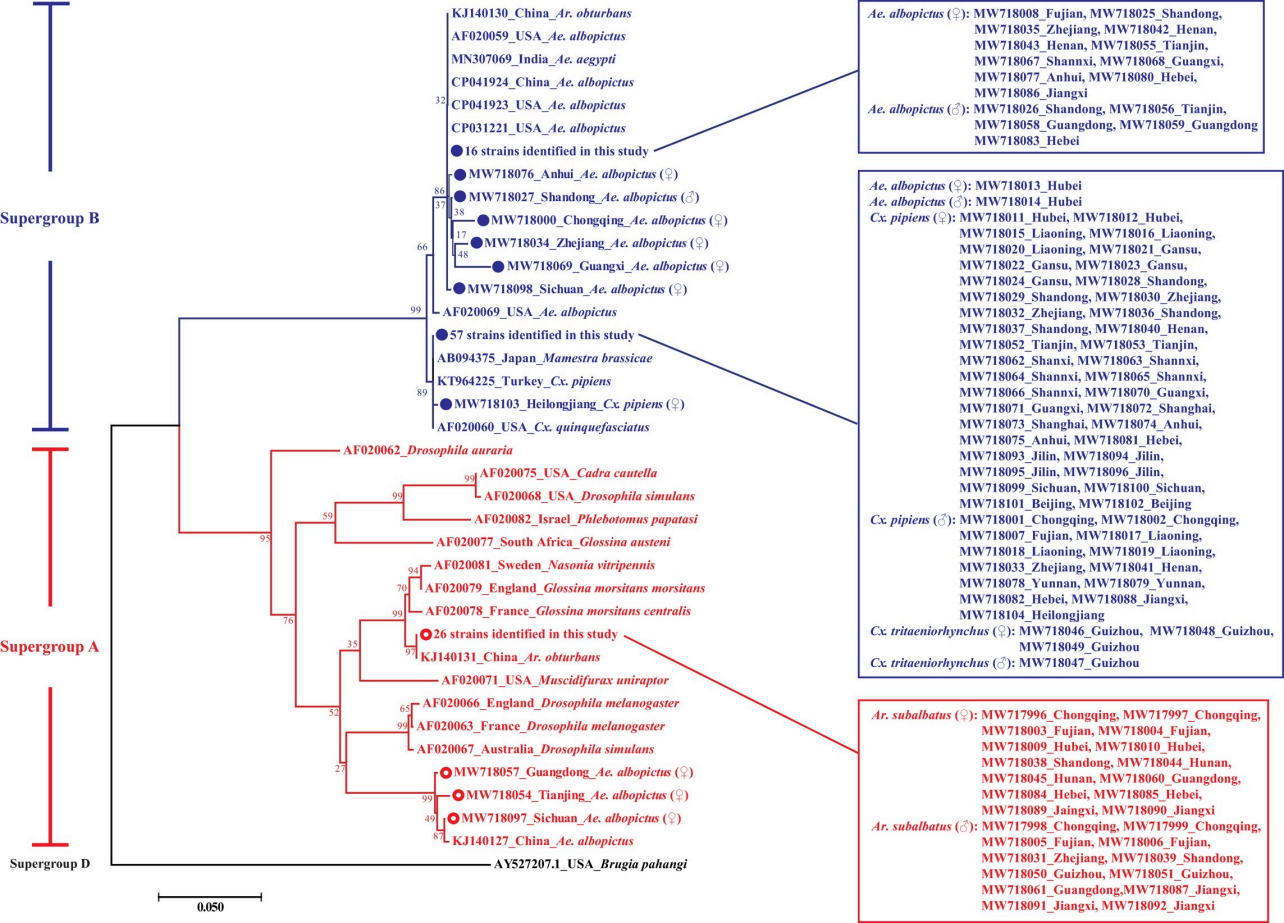
Neighbor-joining phylogenetic tree based on *Wolbachia wsp* gene partial sequences (~610 bp) obtained in this study and GenBank database. Strains identified in this study are identified with open circles (○) for supergroup A (in red) and filled circles (●) for supergroup B (in blue). The numbers at the branches show bootstrap support (1000 replicates). The bar at the bottom of the figure denotes distance.

The sequences of partial *Wolbachia wsp* gene identified in this study were submitted to GenBank with the accession numbers of MW717996~MW718104. The neighbor-joining and maximum-likelihood phylogenetic trees both showed that 26.6% (29/109) and 73.4% (80/109) of the sequenced samples were clustered in supergroup A and B, respectively (Figs 2 and S2). The *Wolbachia* was available to be detected in four species of mosquitoes, including *Ae*. *albopictus*, *Ar*. *subalbatus*, *Cx pipiens* and *Cx*. *tritaeniorhynchus*. Only the positive samples of *Ae*. *albopictus* shared both supergroups, while the *Wolbachia* in *Ar*. *subalbatus* (supergroup A), *Cx*. *pipiens* (supergroup B) and *Cx*. *tritaeniorhynchus* (supergroup B) belonged to a single supergroup, respectively. In supergroup A, the partial sequences of *wsp* gene amplified from 26 *Ar*. *subalbatus* (14 females and 12 males) were identical to KJ140131 identified in *Ae*. *albopictus* in China. The remaining three strains (MW718054, MW718057 and MW718097) in supergroup A were closest to KJ140127 (*Ae*. *albopictus*, China). In supergroup B, the *Wolbachia wsp* sequences identified in this study were clustered into two sub-clades. The first sub-clade consisted the 81.5% (22/27) of *Wolbachia* sequences amplified from *Ae*. *albopictus* in this study. The second sub-clade consisted of all the *Wolbachia wsp* sequences obtained from *Cx*. *pipiens* (n = 52) and *Cx*. *tritaeniorhynchus* (n = 4), and two sequences obtained from *Ae*. *albopictus* (Fig 2).

**Fig 3:**
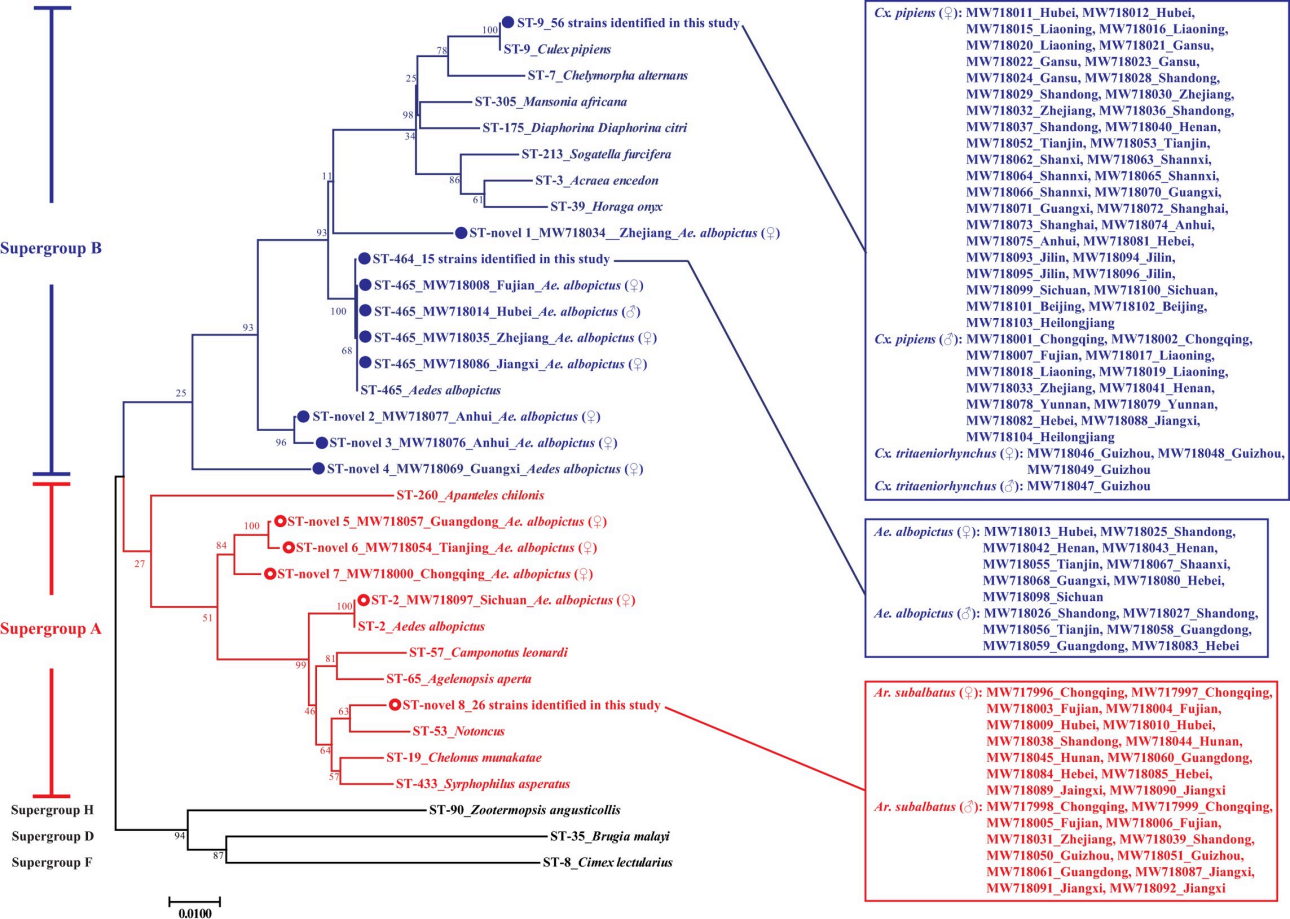
Neighbor-joining phylogenetic tree based on five MLST loci (*gatB*, *coxA*, *hcpA*, *ftsZ* and *fbpA*) of the strains obtained in this study and GenBank database. Strains identified in this study are identified with open circles (○) for supergroup A (in red) and filled circles (●) for supergroup B (in blue). The GenBank accession number of *wsp* sequences of each strain identified in this study was marked on the right side of ST code. The numbers at the branches show bootstrap support (1000 replicates). The bar at the bottom of the figure denotes distance.

Submissions to the MLST database of *Wolbachia* are currently suspended until a new curator can be appointed (https://pubmlst.org/organisms/wolbachia-spp). So for the strains with novel sequence types, we are unavailable to receive the ST codes immediately, and “ST-novel #” was marked in front of these strains instead of the ST codes (Fig 3). There were 12 different sequence types of the *Wolbachia* strains in this study, including four archived sequence types, ST-2 (n = 1), ST-9 (n = 56), ST-464 (n = 15) and ST-465 (n = 4), and eight novel ones (n = 33). The performance of MLST-based phylogenetic tree showed a high consistency with that constructed with *wsp*. Based on MLST, the *Wolbachia* strains identified in this study belonged to supergroups A (n = 30) and B (n = 79). Interestingly, a *Wolbachia* strain identified in an *Ae*. *albopictus* mosquito from Chongqing was clustered into supergroup A and B in the phylogenetic trees of MLST and *wsp*, respectively (Figs 2 and 3). There were three main clusters in both *wsp* and MLST-based phylogenetic trees, and it worth noting that most strains in these clusters corresponded to each other in the two phylogenetic trees (Figs 2 and 3).

### Alignment of nucleotide and deduced amino acid sequences of *wsp* gene

**Fig 4:**
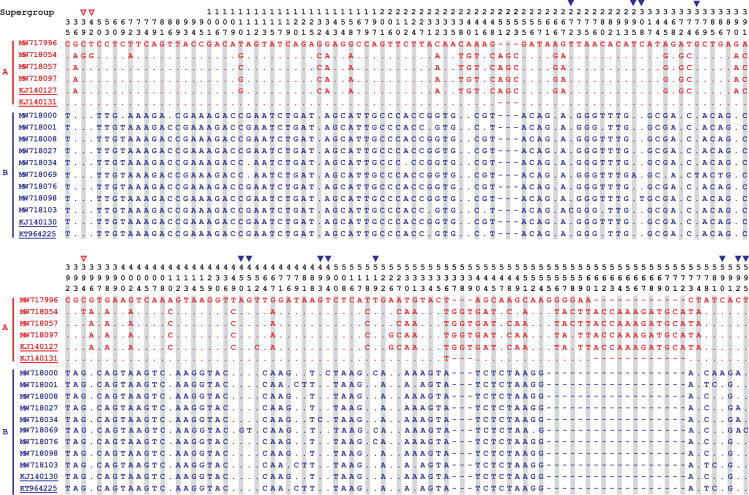
Alignment of partial-length of *Wolbachia wsp* gene nucleotide (~610 bp) sequences identified in this study (MW717996, MW718054, MW718057, MW718097, MW718000, MW718001, MW718008, MW718027, MW718034, MW718069, MW718076, MW718098, MW718103) together with 4 mostly identical reference sequences (KJ140127, KJ140131, KJ140130 and KT964225) obtained in GenBank database. Strains belonging to supergroup A and B are shown in red and blue, respectively, while the reference sequences are indicated with underlines. Numbers, read from top to bottom, above the sequences are nucleotide positions on the *wsp* gene of MW717996. Dots indicate nucleotides identical to the reference sequences. The triangles above nucleotide position numbers indicate sites of mutations in our isolates (supergroup A: ▽ in red, and supergroup B: ▼ in blue) relative to the one or both of the reference sequences.

**Fig 5:**
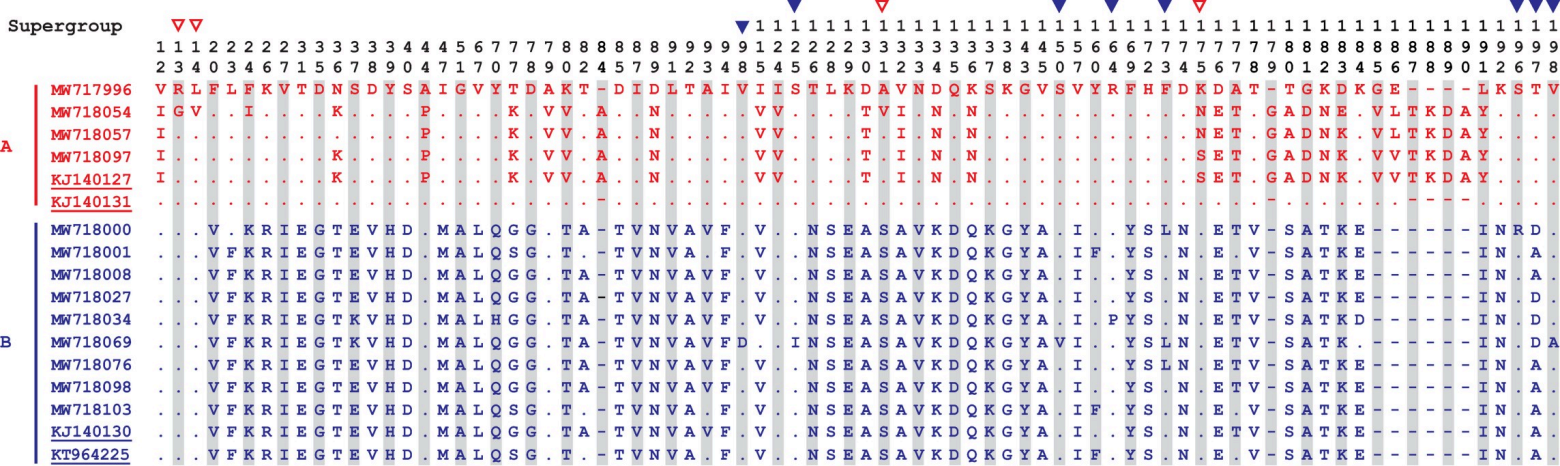
Alignment of partial-length of *Wolbachia wsp* gene deduced amino acid (~200 AA) sequences identified in this study (MW717996, MW718054, MW718057, MW718097, MW718000, MW718001, MW718008, MW718027, MW718034, MW718069, MW718076, MW718098, MW718103) together with 4 mostly identical reference sequences (KJ140127, KJ140131, KJ140130 and KT964225) obtained in GenBank database. Strains belonging to supergroup A and B are shown in red and blue, respectively, while the reference sequences are indicated with underlines. Numbers, read from top to bottom, above the sequences are deduced amino acid positions on the *wsp* gene of MW717996. Dots indicate nucleotides identical to the reference sequences. The triangles above nucleotide position numbers indicate sites of mutations in our isolates (supergroup A: ▽ in red, and supergroup B: ▼ in blue) relative to the one or both of the reference sequences.

When compared with the closest reference sequences (KJ140127, KJ140131, KJ140130 and KT964225) deposited in GenBank, we found that the sequences obtained in this study had a total of 15 unique mutations in partial *wsp* gene. Among them, three mutations (C39G, T42G and C/G394T) were identified from supergroup A and the remaining 12 mutations (T272C, T295A, C308T, G376T, A450G, G451T, G493C, T494C, T519C, C590A, C592A and T595C) were included in supergroup B. Alignment of deduced amino acid sequences demonstrated that there were four (R13G, L14V, A/S131V and K175N/S) and eight (V98D, S125I, S150V, R164P, F173L, S196R, T197D/A and V198A) mutations in supergroups A and B, respectively (Figs 4 and 5).

## Discussion

Approximately 3500 species of mosquitoes belonging to 41 genera are widely distributing in the world, of which at least 350 species have been found in China [22–24], and *Ae*. *albopictus*, *An*. *sinensis*, *Ar*. *subalbatus*, *Cx*. *pipiens* and *Cx*. *tritaeniorhynchus* were dominant populations [25,26]. In this study, we investigated the prevalence of *Wolbachia* among these four genera of mosquitoes captured in 25 provinces of China. *Ae*. *albopictus*, the major vector of arboviruses, is the natural host with a prevalence of almost 100% of *Wolbachia* [13,27,28]. In this study, the total infection rate of *Wolbachia* was 97.1% (331/341) among *Ae*. *albopictus* collected from 17 provinces. The prevalence rates were above 94.7% in most of the sampled provinces, except Guangdong and Liaoning provinces, where the infection rate was significantly lower in the females of *Ae*. *albopictus* in Liaoning province (*P*<0.05). To the best of our knowledge, this study revealed the high prevalence of natural *Wolbachia* infection in *Ar*. *subalbatus* (95.8%, 481/502), *Cx*. *pipiens* (87.0%, 1525/1752) and *Cx*. *tritaeniorhynchus* (17.1%, 14/82) in vast areas of China for the first time (Fig 1, Tables 2 and 3). A few studies have demonstrated the presence of natural *Wolbachia* infection in *Ar*. *subalbatus* [29–31], however, the size and geographical diversity of samples were very limited. In this study, a total of 502 mosquitoes of *Ar*. *subalbatus* were collected in ten provinces, locating in eastern (Fujian, Jiangxi, Shandong and Zhejiang provinces), northern (Hebei province), northeastern (Liaoning province), central (Hubei and Hunan provinces), southwestern (Chongqing and Guizhou provinces) and southern (Guangdong province) of China. The overall infected rate of *Wolbachia* in *Ar*. *subalbatus* in this study (95.8%) is similar to the previous epidemiological survey conducted in Sri Lanka (100%) [30], indicating that *Ar*. *subalbatus* is the naturally harbor of *Wolbachia* (Fig 1 and Table 2). *Cx*. *pipiens* complex mosquitoes are widely distributed throughout China, including at high altitudes, such as Tibet [16,32,33]. Although considered as an important disease vector, there is little information on the distribution and diversity of *Wolbachia* in this kind of mosquitoes in China. In this study, samples of *Cx*. *pipiens* mosquitoes were identified in 23 out of 25 sampled provinces (except for Guizhou and Hunan provinces). The prevalence of *Wolbachia* varied greatly among these mosquitoes from different provinces. For example, only one mosquito of *Cx*. *pipiens* (1.4%, 1/71) was infected in Guangdong province, while there were 100% positive rates in Chongqing, Henan, Liaoning, Shanxi, Sichuan and Yunnan provinces (Fig 1, Tables 2 and 3). We noticed that *Cx*. *pipiens* is not a native mosquito species in Guangdong [15]. Was there a research group that artificially released mosquitoes, resulting in a low rate of *Wolbachia* infection? *Cx*. *tritaeniorhynchus* was only identified in the samples from Guizhou province. Our results showed a prevalence of 17.1% for *Wolbachia* in natural populations of *Cx*. *tritaeniorhynchus* in Guizhou province, China, considerably lower than the positive rates of *Ae*. *albopictus*, *Ar*. *subalbatus* and *Cx*. *pipiens* (Fig 1 and Table 2). For decades, *Anopheles* mosquitoes were considered resistant to *Wolbachia*. Although have been experimental infected in the laboratory, natural infections of *Wolbachia* in wild-collected *Anopheles* mosquitoes have barely been reported [34,35]. *Anopheles* mosquitoes were always considered to be exempt of *Wolbachia* until some recent studies showed evidence of natural *Wolbachia* infections in field populations of several *Anopheles* mosquitoes (*An*. *arabiensis*, *An*. *coluzzii*, *An*. *demeilloni*, *An*. *funestus*, *An*. *gambiae* and *An*. *moucheti*) targeting *16S rRNA* [36–40], but not *wsp* gene. In addition, Walker et al. determined a heavy infection of *Wolbachia* in the ovaries of *An*. *moucheti* that could induce maternal transmission [41]. However, in this study, the mosquitoes of *An*. *sinensis* sampled from ten provinces in this study were all negative for *Wolbachia* by the screening of *wsp* and *16S rRNA* genes (Table 2).

The temporal distribution of *Wolbachia* in arthropods is largely unknown. In this study, mosquitoes were captured at the first day of every month from September 2013 to August 2014, except for January and February, when the temperature was below zero degrees Celsius in average. Although the information on the species and genders of the captured mosquitoes was only identified and noted from April to August, 2014, it was available for us to find a significant reduction of the infected rates of *Wolbachia* in *Cx*. *pipiens* in July (*P*<0.01), in both females and males. Previous studies have indicated that the rearing temperature have significant effects on the fertility, frequency and density of mosquitoes and other arthropods infected with *Wolbachia*, especially *w*Mel and *w*AlbB [42–47]. Further studies are needed to determine the effect on the infection of *Wolbachia* at more extreme temperature conditions (Table 3).

Although do not perform particularly well in the differentiation of closely related *Wolbachia* genomes [8], compared with *16S rRNA* and cell division protein gene (*ftsZ*), phylogenetic tree of improved resolution can be constructed with *wsp* gene, which is evolving at a much faster rate [11]. Molecular phylogeny based on *wsp* sequences revealed that most *Wolbachia* infection in *Ae*. *albopictus* clustered with *w*AlbB (supergroup B) (88.9%, 24/27), while 11.1% (3/27) belonged to *w*AlbA (supergroup A). The genetic variation was observed within both *w*AlbA and *w*AlbB strains, including some strains sampled from the same location (Figs 2 and 4). Compared with previous studies, our results suggested that *Wolbachia* could have invaded and spread throughout populations of these mosquitoes for a long time [48–51]. The information of the diversity of *Wolbachia* infection in *Ar*. *subalbatus* is quite limited. Nugapola et al. reported the presence of *Wolbachia* supergroup A in two mosquitoes in Sri Lanka in 2017 [30]. To the best of our knowledge, this was the only published study regarding the diversity of *Wolbachia* infection in *Ar*. *subalbatus*, until we demonstrated the presence of *Wolbachia* supergroup A in 11 provinces of China. Interestingly, no polymorphism was observed among these strains based on both *wsp* and MLST loci (Figs 2 and 3). In *Cx*. *pipiens*, 98.1% (51/52) of samples were infected with *w*Pip with non-polymorphism based on *wsp* sequence, except for an individual collected from Heilongjiang province (Fig 2). Further studies are needed to investigate the diversity of *w*Pip with higher-resolution molecular biological assays, for example, whole-genome typing methods, more accurate markers and PCR-restriction fragment length polymorphism (RFLP) [8,52]. Bleidorn and Gerth have provided a characterization of 252 single copy loci for the application of few loci approaches, and some of these loci have the potential to perform better than *wsp* and MLST. However, some of these loci may not be harbored by mosquitoes or have no polymorphism in mosquitoes. Further studies are still needed to screen accurate markers from these loci. Although *Rickettsia* were screened in *Cx*. *tritaeniorhynchus* with a low prevalence [53], this mosquito specie was not considered to harbor natural *Wolbachia* infections [54,55]. Interestingly, in this study, *Wolbachia*-positive samples were detected in *Cx*. *tritaeniorhynchus* mosquitoes in Guizhou province and identical with *w*Pip strains (Figs 1–3, S2 and Table 2). Although there were a few outliers, molecular phylogeny based on five MLST loci was generally consistent with that of *wsp* (Figs 2 and 3).

The present study identified a total of 12 unique mutations of the deduced amino acid (AA) with *wsp* gene sequencing and aligning, including four mutations in *Wolbachia* supergroup A and eight mutations in supergroup B, respectively. Two insertions (G_179_ and TKDA_187-190_) were identified among the three strains (MW718054, MW718057 and MW718097) of *Wolbachia* supergroup A. Both the phylogenetic tree and nucleotide/ AA alignment showed that these three strains were mostly closed to the reference sequence (KJ140127) with low polymorphism, although the mosquito vectors were sampled from three far separate provinces (Tianjin, Guangdong and Sichuan provinces) (Figs 1, 2, 4 and 5).

## Supporting information

S1 Fig The sensitivity and specify of *wsp*-based PCR in this study. The 10-fold dilutions were performed to give solutions containing 100,000, 10,000, 1,000, 100 and 10 gene copies per PCR reaction system. The DNA samples of *Rickettsia bellii* and *R*. *felis* and double-distilled water were served as negative controls.
(TIF)

S2 Fig Maximum-likelihood phylogenetic tree based on *Wolbachia wsp* gene partial sequences (~610 bp) obtained in this study and GenBank database. Strains identified in this study are identified with open circles (○) for supergroup A and filled circles (●) for supergroup B. The numbers at the branches show bootstrap support (1000 replicates). The bar at the bottom of the figure denotes distance.
(TIF)

## References

[1] Hertig M, Wolbach SB. Studies on Rickettsia-Like Micro-Organisms in Insects. J Med Res. 1924;44(3):329–74 7. ; PubMed Central PMCID: PMC2041761. 19972605 PMC2041761

[2] Stouthamer R, Breeuwer JA, Hurst GD. Wolbachia pipientis: microbial manipulator of arthropod reproduction. Annu Rev Microbiol. 1999;53:71–102. doi: 10.1146/annurev.micro.53.1.71 . 10547686

[3] Turelli M, Hoffmann AA. Microbe-induced cytoplasmic incompatibility as a mechanism for introducing transgenes into arthropod populations. Insect Mol Biol. 1999;8(2):243–55. doi: 10.1046/j.1365-2583.1999.820243.x . 10380108

[4] McMeniman CJ, Lane RV, Cass BN, Fong AW, Sidhu M, Wang YF, et al. Stable introduction of a life-shortening Wolbachia infection into the mosquito Aedes aegypti. Science. 2009;323(5910):141–4. doi: 10.1126/science.1165326 . 19119237

[5] Jiggins FM. The spread of Wolbachia through mosquito populations. PLoS Biol. 2017;15(6):e2002780. doi: 10.1371/journal.pbio.2002780 ; PubMed Central PMCID: PMC5453404. 28570608 PMC5453404

[6] van den Hurk AF, Hall-Mendelin S, Pyke AT, Frentiu FD, McElroy K, Day A, et al. Impact of Wolbachia on infection with chikungunya and yellow fever viruses in the mosquito vector Aedes aegypti. PLoS Negl Trop Dis. 2012;6(11):e1892. doi: 10.1371/journal.pntd.0001892 ; PubMed Central PMCID: PMC3486898. 23133693 PMC3486898

[7] Bian G, Joshi D, Dong Y, Lu P, Zhou G, Pan X, et al. Wolbachia invades Anopheles stephensi populations and induces refractoriness to Plasmodium infection. Science. 2013;340(6133):748–51. doi: 10.1126/science.1236192 . 23661760

[8] Bleidorn C, Gerth M. A critical re-evaluation of multilocus sequence typing (MLST) efforts in Wolbachia. FEMS Microbiol Ecol. 2018;94(1). doi: 10.1093/femsec/fix163 . 29186405

[9] Laidoudi Y, Levasseur A, Medkour H, Maaloum M, Ben Khedher M, Sambou M, et al. An Earliest Endosymbiont, Wolbachia massiliensis sp. nov., Strain PL13 from the Bed Bug (Cimex hemipterus), Type Strain of a New Supergroup T. International journal of molecular sciences. 2020;21(21). doi: 10.3390/ijms21218064 ; PubMed Central PMCID: PMC7662661. 33138055 PMC7662661

[10] O’Neill SL, Giordano R, Colbert AM, Karr TL, Robertson HM. 16S rRNA phylogenetic analysis of the bacterial endosymbionts associated with cytoplasmic incompatibility in insects. Proc Natl Acad Sci U S A. 1992;89(7):2699–702. doi: 10.1073/pnas.89.7.2699 ; PubMed Central PMCID: PMC48729. 1557375 PMC48729

[11] Zhou W, Rousset F, O’Neil S. Phylogeny and PCR-based classification of Wolbachia strains using wsp gene sequences. Proc Biol Sci. 1998;265(1395):509–15. doi: 10.1098/rspb.1998.0324 ; PubMed Central PMCID: PMC1688917. 9569669 PMC1688917

[12] Baldo L, Dunning Hotopp JC, Jolley KA, Bordenstein SR, Biber SA, Choudhury RR, et al. Multilocus sequence typing system for the endosymbiont Wolbachia pipientis. Appl Environ Microbiol. 2006;72(11):7098–110. doi: 10.1128/AEM.00731-06 ; PubMed Central PMCID: PMC1636189. 16936055 PMC1636189

[13] Hu Y, Xi Z, Liu X, Wang J, Guo Y, Ren D, et al. Identification and molecular characterization of Wolbachia strains in natural populations of Aedes albopictus in China. Parasites & vectors. 2020;13(1):28. doi: 10.1186/s13071-020-3899-4 ; PubMed Central PMCID: PMC6961339. 31937373 PMC6961339

[14] Huang EYY, Wong AYP, Lee IHT, Qu Z, Yip HY, Leung CW, et al. Infection patterns of dengue, Zika and endosymbiont Wolbachia in the mosquito Aedes albopictus in Hong Kong. Parasites & vectors. 2020;13(1):361. doi: 10.1186/s13071-020-04231-x ; PubMed Central PMCID: PMC7372788. 32690078 PMC7372788

[15] Atoni E, Zhao L, Hu C, Ren N, Wang X, Liang M, et al. A dataset of distribution and diversity of mosquito-associated viruses and their mosquito vectors in China. Sci Data. 2020;7(1):342. doi: 10.1038/s41597-020-00687-9 ; PubMed Central PMCID: PMC7555486. 33051449 PMC7555486

[16] Zhang J, Lu G, Li J, Kelly P, Li M, Wang J, et al. Molecular Detection of Rickettsia felis and Rickettsia bellii in Mosquitoes. Vector Borne Zoonotic Dis. 2019;19(11):802–9. doi: 10.1089/vbz.2019.2456 . 31306085

[17] Zhang J, Lu G, Kelly PJ, Wang C. Seasonal and Gender Differences in Presence of Rickettsia felis and Blood meals Provide Additional Evidence of a Vector Role for Mosquitoes. Can J Infect Dis Med Microbiol. 2019;2019:8543460. doi: 10.1155/2019/8543460 ; PubMed Central PMCID: PMC6481102. 31093308 PMC6481102

[18] Folmer O, Black M, Hoeh W, Lutz R, Vrijenhoek R. DNA primers for amplification of mitochondrial cytochrome c oxidase subunit I from diverse metazoan invertebrates. Mol Mar Biol Biotechnol. 1994;3(5):294–9. . 7881515

[19] Werren JH, Windsor DM. Wolbachia infection frequencies in insects: evidence of a global equilibrium? Proc Biol Sci. 2000;267(1450):1277–85. doi: 10.1098/rspb.2000.1139 ; PubMed Central PMCID: PMC1690679. 10972121 PMC1690679

[20] Braig HR, Zhou W, Dobson SL, O’Neill SL. Cloning and characterization of a gene encoding the major surface protein of the bacterial endosymbiont Wolbachia pipientis. J Bacteriol. 1998;180(9):2373–8. doi: 10.1128/JB.180.9.2373-2378.1998 ; PubMed Central PMCID: PMC107178. 9573188 PMC107178

[21] Yang Y, Chu S, Shang S, Yang Z, Wang C. Short communication: Genotyping and single nucleotide polymorphism analysis of bovine leukemia virus in Chinese dairy cattle. J Dairy Sci. 2019;102(4):3469–73. doi: 10.3168/jds.2018-15481 . 30712932

[22] Xia H, Wang Y, Atoni E, Zhang B, Yuan Z. Mosquito-Associated Viruses in China. Virol Sin. 2018;33(1):5–20. doi: 10.1007/s12250-018-0002-9 ; PubMed Central PMCID: PMC5866263. 29532388 PMC5866263

[23] Wang G, Li C, Guo X, Xing D, Dong Y, Wang Z, et al. Identifying the main mosquito species in China based on DNA barcoding. PLoS One. 2012;7(10):e47051. doi: 10.1371/journal.pone.0047051 ; PubMed Central PMCID: PMC3468562. 23071708 PMC3468562

[24] Guo Y, Song Z, Luo L, Wang Q, Zhou G, Yang D, et al. Molecular evidence for new sympatric cryptic species of Aedes albopictus (Diptera: Culicidae) in China: A new threat from Aedes albopictus subgroup? Parasites & vectors. 2018;11(1):228. doi: 10.1186/s13071-018-2814-8 ; PubMed Central PMCID: PMC5885320. 29618379 PMC5885320

[25] Zheng X, Zhong D, He Y, Zhou G. Seasonality modeling of the distribution of Aedes albopictus in China based on climatic and environmental suitability. Infect Dis Poverty. 2019;8(1):98. doi: 10.1186/s40249-019-0612-y ; PubMed Central PMCID: PMC6889612. 31791409 PMC6889612

[26] Zhu G, Xia H, Zhou H, Li J, Lu F, Liu Y, et al. Susceptibility of Anopheles sinensis to Plasmodium vivax in malarial outbreak areas of central China. Parasites & vectors. 2013;6:176. doi: 10.1186/1756-3305-6-176 ; PubMed Central PMCID: PMC3695883. 23768077 PMC3695883

[27] Das B, Satapathy T, Kar SK, Hazra RK. Genetic structure and Wolbachia genotyping in naturally occurring populations of Aedes albopictus across contiguous landscapes of Orissa, India. PLoS One. 2014;9(4):e94094. doi: 10.1371/journal.pone.0094094 ; PubMed Central PMCID: PMC3979767. 24714653 PMC3979767

[28] Kitrayapong P, Baimai V, O’Neill SL. Field prevalence of Wolbachia in the mosquito vector Aedes albopictus. Am J Trop Med Hyg. 2002;66(1):108–11. doi: 10.4269/ajtmh.2002.66.108 . 12135259

[29] Jamnongluk W, Kittayapong P, Baisley KJ, O’Neill SL. Wolbachia infection and expression of cytoplasmic incompatibility in Armigeres subalbatus (Diptera: Culicidae). J Med Entomol. 2000;37(1):53–7. doi: 10.1603/0022-2585-37.1.53 . 15218907

[30] Nugapola N, De Silva W, Karunaratne S. Distribution and phylogeny of Wolbachia strains in wild mosquito populations in Sri Lanka. Parasites & vectors. 2017;10(1):230. doi: 10.1186/s13071-017-2174-9 ; PubMed Central PMCID: PMC5424329. 28490339 PMC5424329

[31] Tsai KH, Lien JC, Huang CG, Wu WJ, Chen WJ. Molecular (sub) grouping of endosymbiont Wolbachia infection among mosquitoes of Taiwan. J Med Entomol. 2004;41(4):677–83. doi: 10.1603/0022-2585-41.4.677 . 15311460

[32] Wang ZM, Li CX, Xing D, Yu YH, Liu N, Xue RD, et al. Detection and widespread distribution of sodium channel alleles characteristic of insecticide resistance in Culex pipiens complex mosquitoes in China. Med Vet Entomol. 2012;26(2):228–32. doi: 10.1111/j.1365-2915.2011.00985.x . 22070231

[33] Liu X, Baimaciwang, Wu H, Pengcuociren, Guo Y, Cirenwangla, et al. Breeding Site Characteristics and Associated Factors of Culex pipiens Complex in Lhasa, Tibet, P. R. China. International journal of environmental research and public health. 2019;16(8). doi: 10.3390/ijerph16081407 ; PubMed Central PMCID: PMC6517927. 31003560 PMC6517927

[34] Joshi D, Pan X, McFadden MJ, Bevins D, Liang X, Lu P, et al. The Maternally Inheritable Wolbachia wAlbB Induces Refractoriness to Plasmodium berghei in Anopheles stephensi. Front Microbiol. 2017;8:366. doi: 10.3389/fmicb.2017.00366 ; PubMed Central PMCID: PMC5340780. 28337184 PMC5340780

[35] Gomes FM, Hixson BL, Tyner MDW, Ramirez JL, Canepa GE, Alves ESTL, et al. Effect of naturally occurring Wolbachia in Anopheles gambiae s.l. mosquitoes from Mali on Plasmodium falciparum malaria transmission. Proc Natl Acad Sci U S A. 2017;114(47):12566–71. doi: 10.1073/pnas.1716181114 ; PubMed Central PMCID: PMC5703331. 29114059 PMC5703331

[36] Baldini F, Segata N, Pompon J, Marcenac P, Shaw WR, Dabire RK, et al. Evidence of natural Wolbachia infections in field populations of Anopheles gambiae. Nat Commun. 2014;5:3985. doi: 10.1038/ncomms4985 ; PubMed Central PMCID: PMC4059924. 24905191 PMC4059924

[37] Niang EHA, Bassene H, Makoundou P, Fenollar F, Weill M, Mediannikov O. First report of natural Wolbachia infection in wild Anopheles funestus population in Senegal. Malar J. 2018;17(1):408. doi: 10.1186/s12936-018-2559-z ; PubMed Central PMCID: PMC6219158. 30400987 PMC6219158

[38] Wong ML, Liew JWK, Wong WK, Pramasivan S, Mohamed Hassan N, Wan Sulaiman WY, et al. Natural Wolbachia infection in field-collected Anopheles and other mosquito species from Malaysia. Parasites & vectors. 2020;13(1):414. doi: 10.1186/s13071-020-04277-x ; PubMed Central PMCID: PMC7425011. 32787974 PMC7425011

[39] Shaw WR, Marcenac P, Childs LM, Buckee CO, Baldini F, Sawadogo SP, et al. Wolbachia infections in natural Anopheles populations affect egg laying and negatively correlate with Plasmodium development. Nat Commun. 2016;7:11772. doi: 10.1038/ncomms11772 ; PubMed Central PMCID: PMC4895022. 27243367 PMC4895022

[40] Jeffries CL, Lawrence GG, Golovko G, Kristan M, Orsborne J, Spence K, et al. Novel Wolbachia strains in Anopheles malaria vectors from Sub-Saharan Africa. Wellcome Open Res. 2018;3:113. doi: 10.12688/wellcomeopenres.14765.2 ; PubMed Central PMCID: PMC6234743. 30483601 PMC6234743

[41] Walker T, Quek S, Jeffries CL, Bandibabone J, Dhokiya V, Bamou R, et al. Stable high-density and maternally inherited Wolbachia infections in Anopheles moucheti and Anopheles demeilloni mosquitoes. Curr Biol. 2021;31(11):2310–20 e5. doi: 10.1016/j.cub.2021.03.056 ; PubMed Central PMCID: PMC8210651. 33857432 PMC8210651

[42] Ant TH, Herd CS, Geoghegan V, Hoffmann AA, Sinkins SP. The Wolbachia strain wAu provides highly efficient virus transmission blocking in Aedes aegypti. PLoS Pathog. 2018;14(1):e1006815. doi: 10.1371/journal.ppat.1006815 ; PubMed Central PMCID: PMC5784998. 29370307 PMC5784998

[43] Lau MJ, Ross PA, Hoffmann AA. Infertility and fecundity loss of Wolbachia-infected Aedes aegypti hatched from quiescent eggs is expected to alter invasion dynamics. PLoS Negl Trop Dis. 2021;15(2):e0009179. doi: 10.1371/journal.pntd.0009179 ; PubMed Central PMCID: PMC7909672. 33591971 PMC7909672

[44] Lau MJ, Ross PA, Endersby-Harshman NM, Hoffmann AA. Impacts of Low Temperatures on Wolbachia (Rickettsiales: Rickettsiaceae)-Infected Aedes aegypti (Diptera: Culicidae). J Med Entomol. 2020;57(5):1567–74. doi: 10.1093/jme/tjaa074 ; PubMed Central PMCID: PMC7566743. 32307514 PMC7566743

[45] Nazni WA, Hoffmann AA, NoorAfizah A, Cheong YL, Mancini MV, Golding N, et al. Establishment of Wolbachia Strain wAlbB in Malaysian Populations of Aedes aegypti for Dengue Control. Curr Biol. 2019;29(24):4241–8 e5. doi: 10.1016/j.cub.2019.11.007 ; PubMed Central PMCID: PMC6926472. 31761702 PMC6926472

[46] Ross PA, Axford JK, Yang Q, Staunton KM, Ritchie SA, Richardson KM, et al. Heatwaves cause fluctuations in wMel Wolbachia densities and frequencies in Aedes aegypti. PLoS Negl Trop Dis. 2020;14(1):e0007958. doi: 10.1371/journal.pntd.0007958 ; PubMed Central PMCID: PMC6977724. 31971938 PMC6977724

[47] Zhu YX, Song ZR, Zhang YY, Hoffmann AA, Hong XY. Spider Mites Singly Infected With Either Wolbachia or Spiroplasma Have Reduced Thermal Tolerance. Front Microbiol. 2021;12:706321. doi: 10.3389/fmicb.2021.706321 ; PubMed Central PMCID: PMC8292952. 34305877 PMC8292952

[48] Armbruster P, Damsky WE Jr., Giordano R, Birungi J, Munstermann LE, Conn JE. Infection of New- and Old-World Aedes albopictus (Diptera: Culicidae) by the intracellular parasite Wolbachia: implications for host mitochondrial DNA evolution. J Med Entomol. 2003;40(3):356–60. doi: 10.1603/0022-2585-40.3.356 . 12943116

[49] Kambhampati S, Rai KS, Burgun SJ. Unidirectional Cytoplasmic Incompatibility in the Mosquito, Aedes Albopictus. Evolution. 1993;47(2):673–7. doi: 10.1111/j.1558-5646.1993.tb02121.x . 28568710

[50] Sinkins SP, Braig HR, O’Neill SL. Wolbachia superinfections and the expression of cytoplasmic incompatibility. Proc Biol Sci. 1995;261(1362):325–30. doi: 10.1098/rspb.1995.0154 . 8587875

[51] de Albuquerque AL, Magalhaes T, Ayres CF. High prevalence and lack of diversity of Wolbachia pipientis in Aedes albopictus populations from Northeast Brazil. Mem Inst Oswaldo Cruz. 2011;106(6):773–6. doi: 10.1590/s0074-02762011000600021 . 22012236

[52] Dumas E, Atyame CM, Milesi P, Fonseca DM, Shaikevich EV, Unal S, et al. Population structure of Wolbachia and cytoplasmic introgression in a complex of mosquito species. BMC Evol Biol. 2013;13:181. doi: 10.1186/1471-2148-13-181 ; PubMed Central PMCID: PMC3846486. 24006922 PMC3846486

[53] Guo WP, Tian JH, Lin XD, Ni XB, Chen XP, Liao Y, et al. Extensive genetic diversity of Rickettsiales bacteria in multiple mosquito species. Sci Rep. 2016;6:38770. doi: 10.1038/srep38770 ; PubMed Central PMCID: PMC5146937. 27934910 PMC5146937

[54] Jeffries CL, Walker T. The Potential Use of Wolbachia-Based Mosquito Biocontrol Strategies for Japanese Encephalitis. PLoS Negl Trop Dis. 2015;9(6):e0003576. doi: 10.1371/journal.pntd.0003576 ; PubMed Central PMCID: PMC4472807. 26086337 PMC4472807

[55] Tiawsirisup S, Sripatranusorn S, Oraveerakul K, Nuchprayoon S. Distribution of mosquito (Diptera: Culicidae) species and Wolbachia (Rickettsiales: Rickettsiaceae) infections during the bird immigration season in Pathumthani province, central Thailand. Parasitol Res. 2008;102(4):731–5. doi: 10.1007/s00436-007-0825-z . 18066693

