# Supplementary figures and images for: Prevalence and molecular characterization of Wolbachia in field-collected Aedes albopictus, Anopheles sinensis, Armigeres subalbatus, Culex pipiens and Cx. tritaeniorhynchus in China
Source: PLoS Negl Trop Dis. 2021 Oct 28;15(10):e0009911. doi: 10.1371/journal.pntd.0009911 (PMC8577788; doi:10.1371/journal.pntd.0009911)

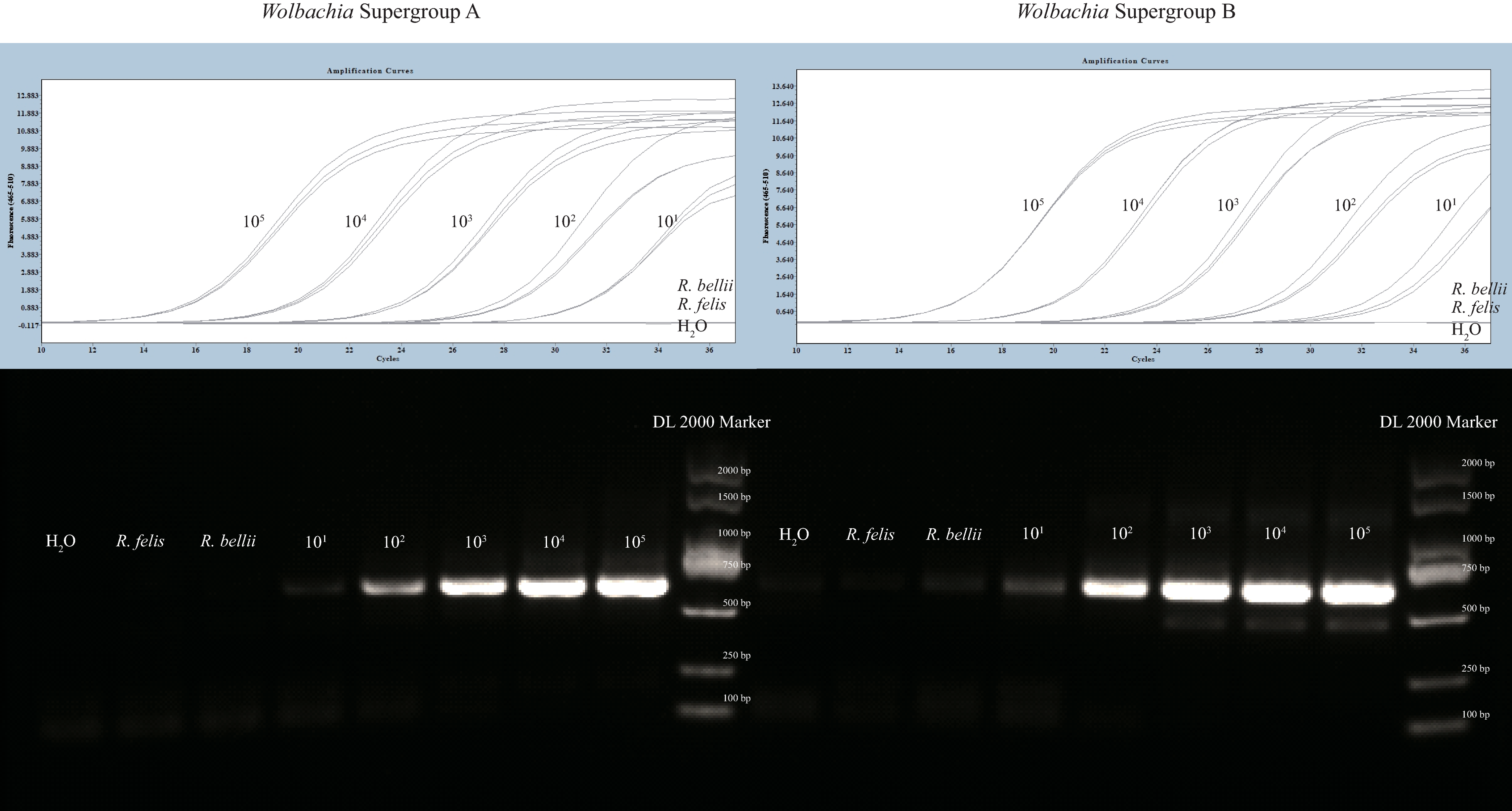

Supplement: S1 Fig — The 10-fold dilutions were performed to give solutions containing 100,000, 10,000, 1,000, 100 and 10 gene copies per PCR reaction system. The DNA samples of Rickettsia bellii and R. felis and double-distilled water were served as negative controls. (TIF) [file pntd.0009911.s001.tif]

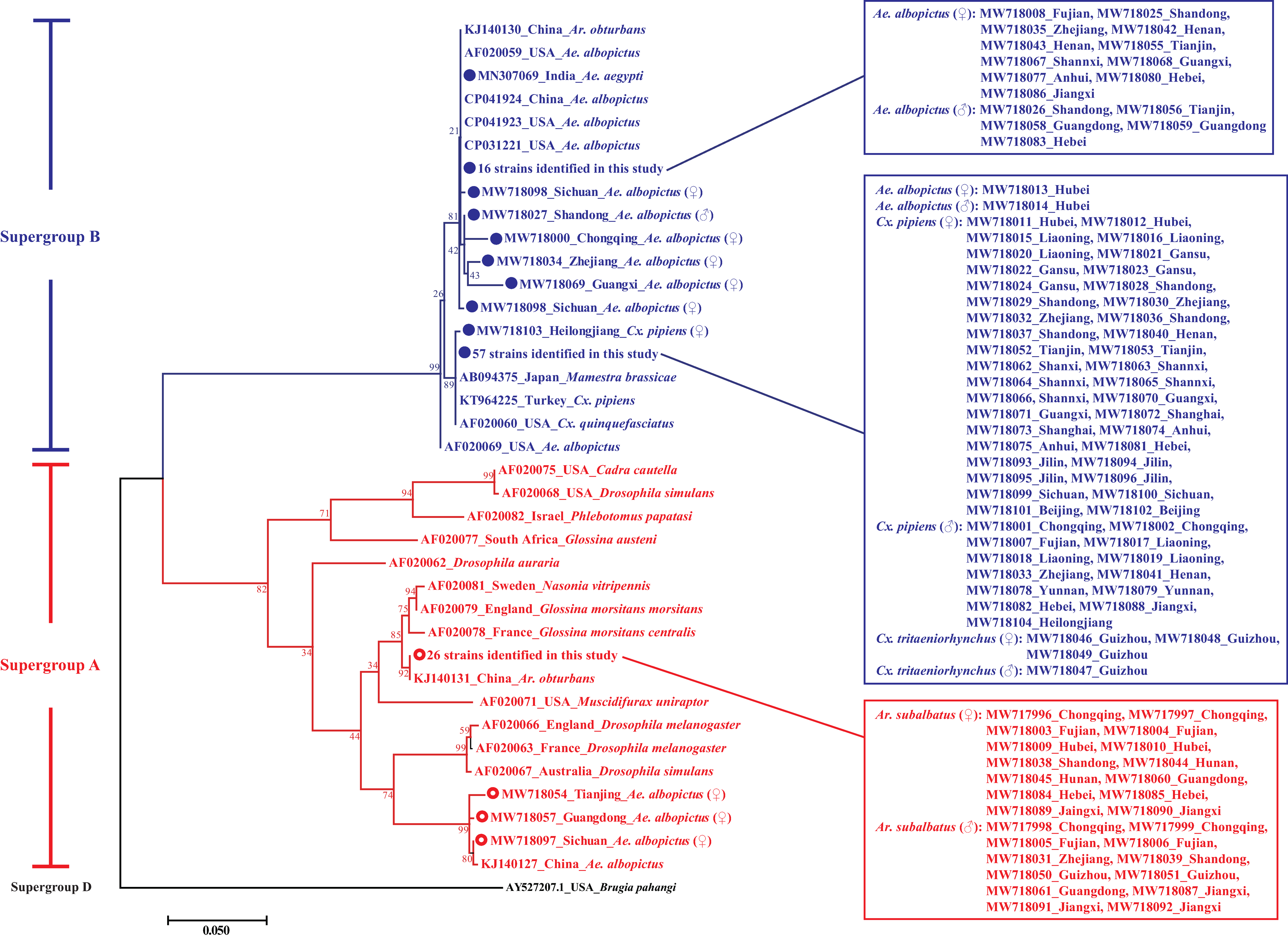

Supplement: S2 Fig — Strains identified in this study are identified with open circles (○) for supergroup A and filled circles (●) for supergroup B. The numbers at the branches show bootstrap support (1000 replicates). The bar at the bottom of the figure denotes distance. (TIF) [file pntd.0009911.s002.tif]
